# Genetic confounding in the association of early motor development with childhood and adolescent exercise behavior

**DOI:** 10.1186/s12966-024-01583-w

**Published:** 2024-03-21

**Authors:** Yahua Zi, Meike Bartels, Conor Dolan, Eco J.C. de Geus

**Affiliations:** 1 School of Exercise and Health, Shanghai University of Sport, Shanghai, China; 2 Department of Biological Psychology, Vrije Universiteit Amsterdam, van der Boechorststraat 7, H541, Medical Faculty Building, Amsterdam, 1081 BT Netherlands; 3 Department of Biological Psychology, Netherlands Twin Register, Vrije Universiteit Amsterdam, Amsterdam, The Netherlands; 4 Amsterdam Public Health Research Institute, Amsterdam University Medical Center, Amsterdam, The Netherlands

**Keywords:** Twin study, Motor milestones, Gross motor skills, Multivariate genetic modeling, Causal modeling

## Abstract

**Introduction:**

Early motor development has been found to be a predictor of exercise behavior in children and adolescents, but whether this reflects a causal effect or confounding by genetic or shared environmental factors remains to be established.

**Methods:**

For 20,911 complete twin pairs from the Netherlands Twin Register a motor development score was obtained from maternal reports on the timing of five motor milestones. During a 12-year follow-up, subsamples of the mothers reported on the twins’ ability to perform seven gross motor skills ability (*N* = 17,189 pairs), and weekly minutes of total metabolic equivalents of task (MET) spent on sports and exercise activities at age 7 (*N* = 3632 pairs), age 10 (*N* = 3735 pairs), age 12 (*N* = 7043 pairs), and age 14 (*N* = 3990 pairs). Multivariate phenotypic and genetic regression analyses were used to establish the predictive strength of the two motor development traits for future exercise behavior, the contribution of genetic and shared environmental factors to the variance in all traits, and the contribution of familial confounding to the phenotypic prediction.

**Results:**

Significant heritability (h^2^) and shared environmental (c^2^) effects were found for early motor development in boys and girls (h^2^ = 43-65%; c^2^ = 16-48%). For exercise behavior, genetic influences increased with age (boys: h^2^_age7_ = 22% to h^2^_age14_ = 51%; girls: h^2^_age7_ = 3% to h^2^_age14_ = 18%) paired to a parallel decrease in the influence of the shared environment (boys: c^2^_age7_ = 68% to c^2^_age14_ = 19%; girls: c^2^_age7_ = 80% to c^2^_age14_ = 48%). Early motor development explained 4.3% (*p* < 0.001) of the variance in future exercise behavior in boys but only 1.9% (*p* < 0.001) in girls. If the effect in boys was due to a causal effect of motor development on exercise behavior, *all* of the factors influencing motor development would, through the causal chain, also influence future exercise behavior. Instead, only the genetic parts of the regression of exercise behavior on motor development were significant. Shared and unique environmental parts of the regression were largely non-significant, which is at odds with the causal hypothesis.

**Conclusion:**

No support was found for a direct causal effect in the association between rapid early motor development on future exercise behavior. In boys, early motor development appears to be an expression of the same genetic factors that underlie the heritability of childhood and early adolescent exercise behavior.

**Supplementary Information:**

The online version contains supplementary material available at 10.1186/s12966-024-01583-w.

## Introduction

Regular exercise behavior in leisure time, such as jogging, exercising at fitness clubs, participation in a recreational or competitive team (e.g. soccer, hockey) and individual (e.g. athletics, tennis, swimming) sports, is now rapidly becoming a major source of daily life physical activity in children and adolescents [1]. This specific domain of physical activity tends to be transmitted from childhood to adulthood [2, 3], improving health-related quality of life and reducing mortality from all causes, including cardiovascular disease [4, 5]. Despite these well-established favorable effects, the majority of children and adolescents do not meet recommended physical activity levels [6, 7], which can have long-term consequences on their health and wellbeing [8, 9].

One factor that has been hypothesized to facilitate regular physical activity and exercise activities in childhood and adolescence is early motor development [10, 11]. Early motor development refers to the acquisition of motor milestones (i.e., turning over, sitting, crawling, standing up and walking) and gross motor skills (i.e., hopping, one-leg standing, throwing, kicking, and catching a ball) during the first few years of life. Delayed motor development is associated with lower physical activity levels in childhood [12]. Stodden and colleagues provided a conceptual model to explain the role of early motor development in children’s physical activity through the mediating effects of perceived motor competence and physical fitness [13]. Extensive testing of various components of this model in the past two decades in many studies have been summarized in a series of reviews and meta-analyses [8, 14–16].

While the current findings from these studies are compelling in their support for the Stodden model, they are largely based on cross-sectional associations, with evidence from long-lasting follow-up studies being more sparse. Even when prospective associations are found, confounding by underlying factors cannot be ruled out. These factors include differences in the early shared (family) environment and differences in genetic make-up. Twin and family studies are in support of a significant role of genetic and shared environmental factors in the regulation of both physical activity behavior [17–32] and early motor development [12, 33–35]. The heritability estimates of physical activity behavior strongly depend on the age of the child, with the shared (family) environment playing the largest role in childhood, but decreasing into adolescence, where genetic factors explain the largest part of variance in physical activity [18, 36]. Substantial heritability estimates have also been reported for early motor milestones achievement (e.g., age of first time being able to sit without support) in a large-scale study (30,256 complete twin pairs) from the Netherlands Twin Register (NTR). In that study, genetic factors explained 52% of the variance in motor milestones achievement [37]. For gross motor skills in young children (e.g., throwing a ball) almost no studies have addressed the role of genetic factors, but a few reports on balancing ability did show substantial heritability (41–62%) [35, 38].

The heritable components in early motor milestone(s) achievement and possibly gross motor skills, on the one hand, and in physical activity (including regular exercise), on the other hand, render plausible the contribution of genetic confounding to the association between early motor development and later childhood and adolescence physical activity. In addition, the impact of the shared family environment, including parental support and attitudes on the importance of both motor milestones and physical activity, could further contribute to this association. Both these confounders may act to diminish the importance of a direct causal effect, which is now prominent in the most established theoretical framework [13]. These familial factors (i.e., genetic and shared environmental factors) may even entirely cause the observed associations between early motor development and later physical activity behaviors. However, to the best of our knowledge, no studies exist on whether early motor development and physical activity share a common genetic and/or environmental background.

To address this gap in our knowledge, we analyzed a very large twin dataset from the NTR to assess the common genetic and/or environmental factors between early motor development based on five important early motor milestones before age 2, and seven gross motor skill items at age 5, on the one hand, and regular exercise behavior during leisure time at age 7, 10, 12, and 14, on the other hand. Although we acknowledge that many physical activity behaviors occur outside of leisure time, the salience of leisure time sports and exercise activities allows them to be reported with much less bias than total physical activity [39]. In addition, leisure time sports and exercise activities are a crucial part of daily physical activity in youngsters, in that they tend to transition into long-term activity habits [40].

The longitudinal nature of the present twin study design was used to estimate the genetic and/or environmental influences that are common to early motor milestone achievement (age 2), gross motor skills (age 5), and later childhood and early adolescent exercise behavior (ages 7–14). In multivariate models we tested whether the hypothesized causal effect of early motor development on future exercise behavior remains plausible after taking genetic and shared environmental confounding into account. Specifically, we tested the causal hypothesis by testing two of the predictions it makes for longitudinal twin data [41, 42]. First, using ‘ordinary’ regression at the phenotypic level we expect to find that higher levels of motor development at ages 2 and 5 will predict higher levels of exercise behavior at follow-up on ages 7 to 14. The absence of a longitudinal association falsifies the causal hypothesis. However, the reverse is not necessarily true. Common underlying factors may independently more rapid early motor development and higher engagement in exercise behaviors, resulting in a longitudinal correlation which may create a false impression of causality. We therefore also tested a second prediction, namely that *all* (observed or latent) factors that influence the hypothesized causal factor (here motor development) will, through the causal chain, also influence the outcomes affected by the causal factors (here future exercise behavior). The twin model allows a direct test of this hypothesis by decomposing the phenotypic regression into its genetic and environmental parts. For any of the genetic, shared environmental and/or unique environmental factors that are found to significantly influence motor development - which can be established in a multivariate twin analysis – we expect to also find regression between early motor development and future exercise behaviors at the level of these genetic, shared, and/or unique environmental factors. Failure to do so falsifies the causal hypothesis.

## Methods

### Participants

The study involved twins born between 1986 and 2016 and registered in the Young NTR (YNTR) study, a large-scale population-based cohort of twins in which a large number of variables are obtained through survey and experimental research [43]. Starting around 1986, NTR systematically approached parents with the request to register their newborn twins in the YNTR and to consent to participate in research on these twins. Recruitment was done through a commercial ‘birth felicitation’ service that delivers a gift box to a large part of all newborns and with the support of the Dutch Society of Parents of Multiples (Nederlandse Vereniging van Ouders van Meerlingen: NVOM; https://www.nvom.nl). Comparison to data from the National Bureau of Statistics records shows that around 40% of the Dutch twin-pairs born from 1986 until 2016 are registered with the NTR (CBS; https://www.cbs.nl/en-gb/figures/detail/37422eng?q=twins/). A detailed composition of the sample in terms of age, sex and zygosity is provided in Supplementary Table 1.

After registration and subject to their informed consent, the mothers received a survey in the months after registration about the course of pregnancy, the twins’ birth, and early developmental characteristics. The first survey included a one-page notebook with a list of motor milestones that mothers were asked to keep track of. After the first survey was returned, the mothers of twins received a second survey including questions about the age at which the twins achieved motor milestones by the age of two [44]. Across three decades, the mean response rate is 65%. When the twins were five years old, both mothers and fathers of twins received a third survey, which contained questions concerning the gross motor development of the twins. Because the correlation between the gross motor scores of mothers and fathers was 0.78 and only the mothers had reported on the motor milestones, we restrict the analysis to mother reports. Across three decades, the mean maternal response rate is 52%. After age 5 up to age 12, extensive surveys on psychological traits, lifestyle, and health were sent every two to three years to the parents. Mean response rates vary from 37 to 47% for the mothers and 26 to 33% for the fathers. Both parents filled out surveys for 70% of the twins; for 27% only the mother and for 3% only the father filled out the survey. In the survey at age 14, the twins themselves reported their sports and exercise activities (response rate 43%). All of the parental and self-report surveys included the comparable questions on the type, amount, and frequency of their regular sports and exercise activities.

For the present analyses, we included the mother’s report on the month in which various motor milestones were achieved (“survey Two”), gross motor development at age 5 (“survey Five”), and the twins’ exercise behavior at age 7 (“survey Seven”), 10 (“survey Ten”), and 12 (“survey Twelve”). We also included the exercise behavior as reported by the twins themselves at age 14 (“survey Fourteen”). We excluded 1572 twins with physical disabilities that likely interfered with their ability to engage in regular physical activity (e.g., hemiplegia, severe scoliosis, ataxia, or missing limbs). To ensure that children were within a similar age range at each data collection wave, twins whose survey data were obtained more than 2 years beyond the target age were excluded. In addition, twins were excluded if information concerning sex or zygosity was missing. Given these exclusion criteria, 20,911 complete twin pairs were available for survey Two, 17,189 for survey Five, 3632 for survey Seven, 3735 for survey Ten, 7043 for survey Twelve, 3990 for survey Fourteen.

The zygosity in the same-sex twins was determined by DNA genotyping in 13.8% (age 2) to 28.8% (age 14) of the twins, or by multiple survey items on physical resemblance and confusion of the twins by family members and others. The accuracy of zygosity determination by survey items has shown 91.8–97.5% agreement with DNA polymorphisms in children aged 2–14 years [43].

### Measures

#### Early motor milestones

A detailed description of the assessment of motor milestones in the NTR can be found elsewhere [12, 37]. Briefly, by the twins’ second birthday, mothers were asked to report the age at which their twins achieved the motor milestones included in the following questions: “At how many months could your [youngest/oldest] twin for the first time roll over from back to belly, sit without support, crawl on hands and knees, stand without support, and walk without support?” About 3.5–8.5% values were missing for multiple motor milestones. When only one single motor milestone was missing, we substituted the missing motor milestone with the sample mean, thus increasing the available sample size. A principal component factor analysis was used to summarize five motor milestones items into a single ‘motor development’ factor score (MD-FS), using an eigenvalue of 1 as the cut-off. Only one factor exceeded the eigenvalue criteria of 1, and this factor explained 61.3% of the variance in the scores of the five items.

#### Gross motor development

In the survey Five, the mothers reported on the twins’ gross motor development when they were five years old. This survey included the question “Can the child…?” followed by the following motor behaviors: “hop more than one time on the same leg”, “stand on one leg longer than 10 seconds”, “throw a ball in a fixed direction”, “kick a ball in a fixed direction”, “catch a ball”, “walk down the staircase without putting both feet on a step at the same time”, and “walk down a staircase without using the handrail?”. The responses were “no” (coded as 0), “yes” (coded as 1), or “not certain” (coded as 0). The response “not certain” was coded as “0”, as we assumed that, if the mothers were not sure about a gross motor skill, the twin likely had not yet attained that skill. Summation across the seven items was used to reduce seven gross motor items into one score (range 0–7) for gross motor development at age 5 (GM5). For 6% of the twins, one or more items were missing. For these twins the gross motor development score was set to missing. For consistency with the coding of the motor milestones achievement (higher MD-FS means later motor milestone achievement), we also reverse coded the gross motor development at age 5 by subtracting the score from the total possible score of seven (higher GM5 means less gross motor skills mastered).

#### Exercise behavior

Exercise and sports activities during leisure time were assessed with consistent measures across surveys, which included comparable questions on the type and amount of twins’ exercise behavior. In survey 7, 10, and 12, mothers of twins were provided a list of common exercise activities in the Netherlands, such as athletics, badminton, ballet/dance, basketball, fitness training, gymnastics, handball, jogging/running, hockey, netball, horseback riding, (ice-skating), tennis, martial arts, soccer, swimming, volleyball, and the option to add additional non-listed activities in free fields. Activities were counted irrespective of whether they were performed competitively or recreationally, or whether performed in a team or solitary. Participation in physical education class and school swimming were separately queried. The questions were: (1) whether the twin participated in the exercise behavior, and, if the answer was “yes”, numerical answers were queried concerning (2) how many years, (3) how many months a year, (4) how many times a week, and (5) how many minutes each time they engaged in the corresponding activity. Adolescents aged 14 reported their own activities in the same way. This study focuses on regular, structured, leisure time exercise behavior, and excluded free play and the physical activities related to school-time (physical education lessons, school swimming), travel, or active transportation (walking, biking), and irregular exercise activities that started less than six months ago, or that were performed for less than three months a year (e.g. skiing).

Exercise behavior was quantified by weekly Metabolic Equivalents of Task (MET score), which is the ratio of the specific activity metabolic rate to the resting metabolic rate (i.e., 1 MET). Each listed activity was assigned a MET score based on the compendium of energy expenditure for youth (aged 6.0–17.9 years) by Ridley et al. [45]. For all the individuals, the product of assigned MET score, weekly frequency, and duration was summed across all the activities to obtain the total weekly METminutes (“MET”) of regular exercise activities during leisure time. Hence, we obtain a weekly total MET spent on leisure-time physical activity at ages 7 (MET7), 10 (MET10), 12 (MET12), and 14 (MET14).

### Statistical analyses

Because the means and variances for both motor development and exercise behavior tend to differ for males and females [31, 37], the analyses were stratified by sex, i.e., conducted separately in the males and females. The male sample consisted of monozygotic (MZ) and dizygotic (DZ) same-sex (male) twin pairs, and the male members of DZ opposite sex (DZOS) twin pairs. The female sample consisted of MZ and DZ female twins and the female members of the DZOS twin pairs. This yields incomplete male and female pairs with only one member of the DZOS twin, but these incomplete pairs were retained because they are informative for the variance of the traits and for the phenotypic associations across traits and across time. The MET scores at all ages were rescaled by subtracting the mean of the MET at age 7 and dividing by the standard deviation of the MET at age 7. The rescaling was done to facilitate optimization of the maximum likelihood estimation function, and to facilitate the specification of starting values. Otherwise this (linear) transformation does not affect the substantive results or conclusions based on the results.

**Fig. 1:**
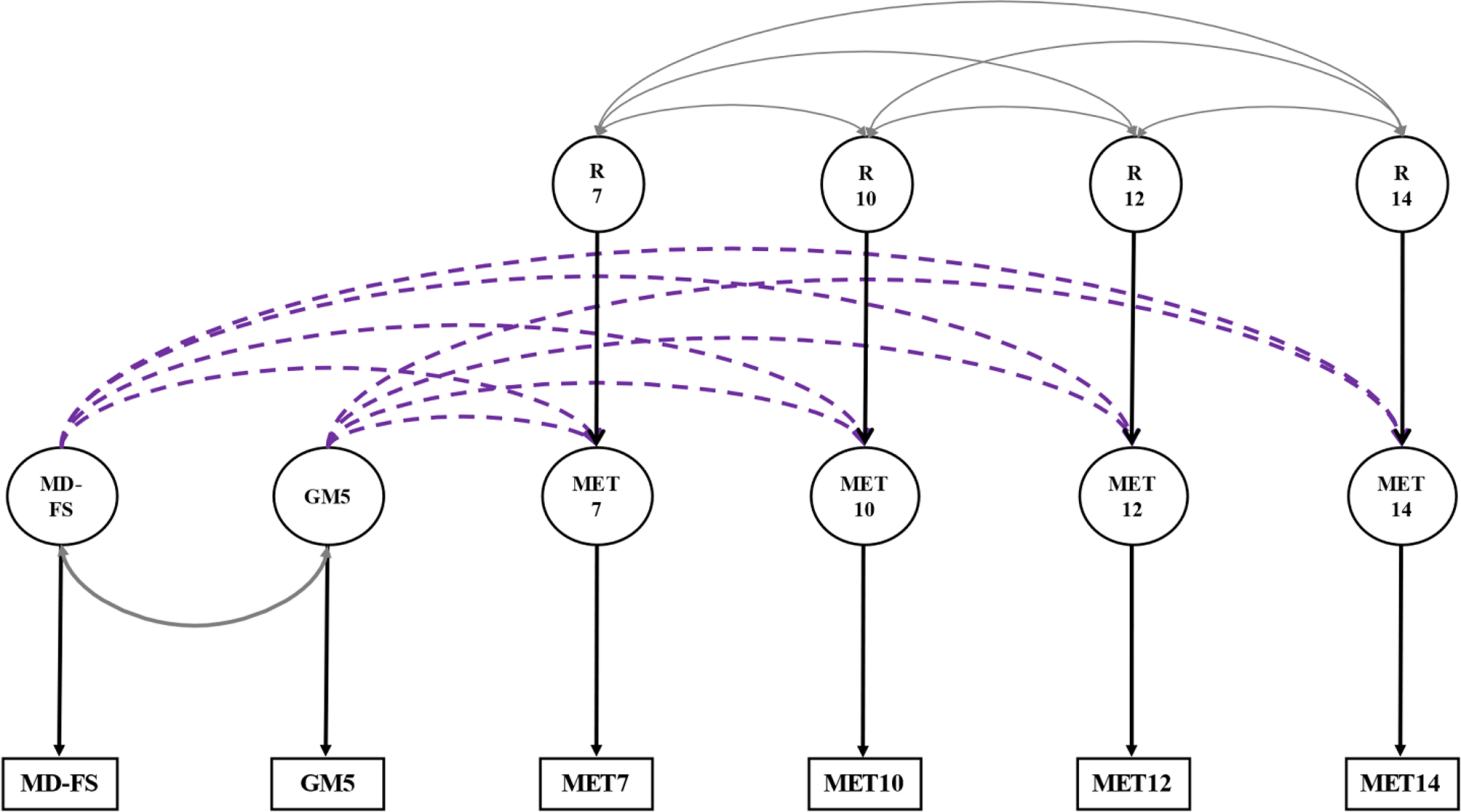
The phenotypic regression model *Note*: Dashed lines between the early motor development traits and the exercise behaviors are single headed arrows denoting the regression relations. Continuous double headed arrows denote the correlations between the two motor development traits and between the residuals of exercise behavior at the various ages. MD-FS, the factor score of motor development before age 2; GM5, gross motor development at age 5; MET7, MET10, MET12, and MET14 represent the voluntary exercise behavior at each age (7, 10, 12, and 14 years); R7, R10, R12, and R14 represent the residuals of the predicted exercise behaviors at each age

*Phenotypic analyses.* To assess the phenotypic associations among the variables, we first computed the 6 × 6 matrices of Pearson correlations. To test whether the two motor development scores (MD-FS and GM5) predicted the later exercise behaviors we conducted multivariable regression analyses based on the model presented in Fig. 1. This model incorporates the tracking of exercise behaviors across time in the correlated residuals.

*Genetic analyses.* Genetic analyses were based on the classical twin design, which capitalized on the fact that MZ twins are genetically identical, whereas DZ twins, like full siblings, share on average 50% of their segregating alleles. The phenotypic resemblance, as quantified by the twin correlation, is attributable to factors shared by family members which include both genetic and shared environmental influences, whereas person-specific or unique environmental factors (including measurement error) only contribute to phenotypic differences among members of twin pairs. We formulated the twin model as a genetic structural equation model to decompose the phenotypic (co)variance into additive genetic (A) and shared (C) environmental, and unique environmental (unshared; E) (co)variance components [46].

**Fig. 2:**
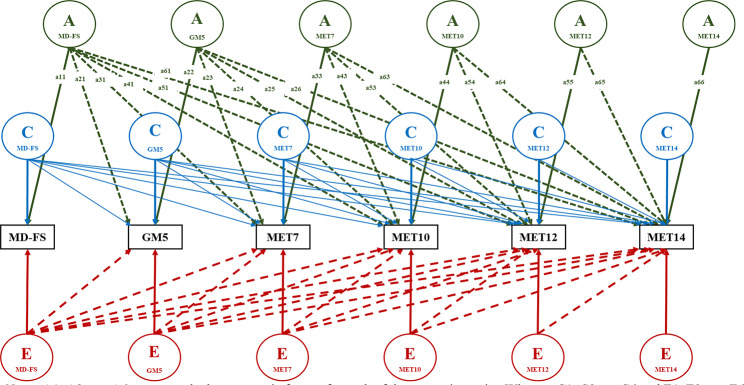
Multivariate ACE Cholesky decomposition *Note*: A1, A2, …, A6 represent the latent genetic factors for each of the respective traits, Whereas C1, C2, … C6 and E1, E2, …, E6 represent the latent shared and unique environmental factors influencing the six traits; The six traits are shown only for one twin, but a symmetric model applies to the co-twin. The latent genetic factors are correlated unity in monozygotic twins and 0.5 in dizygotic twins, whereas the latent shared environmental factors are correlated 1 in both types of twins and the unique environmental factors are correlated 0. Path coefficients in green (---) a11, a21, …, a66 represent loadings of the latent A components on the six traits. Path coefficents for C and E are not drawn to reduce visual clutter but blue (**---**) lines represent the loadings of the shared environmental factors, and red (**---**) lines represent the of the unique environmental factors

Genetic structural equation modeling was done with the OpenMx library [47] version 2.21.1 under R 4.2.3 [48]. Given the presence of missing data we used raw data maximum likelihood estimation. First, we fitted a saturated model which included the 12 × 12 MZ and DZ phenotypic covariance matrices to the data to test (using likelihood ratio tests) the equality of means and variances across birth order, and across MZ and DZ zygosity. Next we fitted an ACE model, using the Cholesky decomposition of the six traits [49] to estimate the 6 × 6 A, C, and E covariance matrices (see Fig. 2). Based on the results, we calculated the relative contributions of A, C, and E to the phenotypic variance of the 6 phenotypes and the A,C, and E correlations among the 6 phenotypes.

**Fig. 3:**
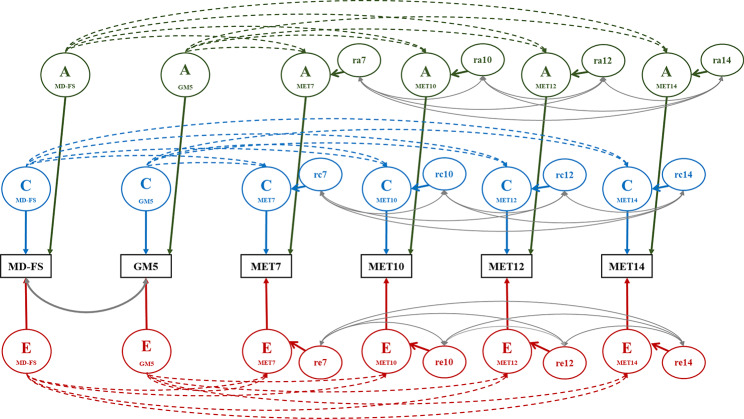
The ACE regression model *Note*: Dashed lines between the latent A, C, and E components for the early motor development traits and for the exercise behaviors are single headed arrows denoting the regression relations. Continuous double headed arrows denote the correlation between the two motor development traits and the correlations between the residuals of the latent A (ra7 - ra14), C (rc7 – rc14), and E (re7 – re14) components for exercise behavior at the various ages. MD-FS, the factor score of motor development before age 2; GM5, gross motor development at age 5; MET7, MET10, MET12, and MET14 represent the voluntary exercise behavior at each age (7, 10, 12, and 14 years)

We proceeded by fitting an ACE regression model (see Fig. 3). Whereas the phenotypic regression model involves predicting the observed dependent MET variables (at ages 7, 10, 12, and 14) from the observed predictors MD-FS and GM5, the ACE regression model involves fitting regression models to the A, C, and E covariance matrices [50]. This allows us to determine the contributions of A, C, and E to the prediction. We explored the role of A, C and E in the prediction by comparing the fit of the ACE regression model to multiple submodels that constrained the regression coefficients to zero. These comparisons were based on the likelihood ratio test. As outlined above, in the presence of significant contributions of A, C, and E to early motor development, the absence of a significant correlations between these A, C, and E components and the A, C, and E components of future exercise behavior falsifies the causal hypothesis, because under the causal model all factors influencing early motor development are expected, through the causal chain, to also be reflected in future exercise behaviors.

## Results

The average age at which the last motor milestone (first time being able to walk without support) was achieved was 15.01 (± 2.43) months. A median of six gross motor skills were mastered at age 5, indicating the majority of the children could perform most of the seven gross motor skills. Table 1 depicts the available complete twin pairs, and the means and standard deviations of the motor development factor scores based on the principal component analysis of the five motor milestones jointly before age 2 (MD-FS, lower values signaling more rapid development), the count of gross motor skills at age 5 (GM5, lower values signaling more rapid development), and the weekly total MET score during leisure time at age 7 (MET 7), 10 (MET 10), 12 (MET 12), and 14 (MET 14) for each zygosity group. As expected, the total MET minutes spent on sports and exercise activities per week during leisure time increased from 7 to 14 years old. Using an estimated intensity of 4 MET for the sports and exercise activities in which the twins engaged, the percentage of the participating children that met the national activity guidelines of 60 min per day of moderate to vigorous activity, was 7% at age 7, 18% at age 10, and 30% at age 12 and 34% at age 14. However, by excluding active play and transportation by biking these percentages are likely an underestimation, particularly at the younger ages.

**Table 1:** Means, standard deviations (SD) of early motor development and later exercise behavior traits

Traits	Total		MZM		DZM		MZF		DZF		DOS	
	Mean (SD)	N	Mean (SD)	N	Mean (SD)	N	Mean (SD)	N	Mean (SD)	N	Mean (SD)	N
MD-FS	0.00 (1.00)	20,911	0.0 (1.0)	3510	0.0 (1.0)	3527	0.0 (1.0)	3724	−0.1 (1.0)	3258	0.0 (1.0)	6874
GM5	0.55 (0.98)	17,189	0.6 (1.0)	2914	0.6 (1.0)	2900	0.4 (0.8)	3161	0.4 (0.9)	2687	0.6 (1.1)	5527
MET7	733 (665)	3632	871 (800)	675	871 (800)	665	536 (514)	618	510 (507)	526	799 (659)	1148
MET10	1120 (867)	3735	1274 (885)	691	1274 (885)	672	824 (679)	651	818 (714)	525	1205 (870)	1196
MET12	1322 (1068)	7043	1104 (1584)	1269	1567 (1104)	1153	944 (863)	1314	947 (860)	1040	1466 (1104)	2267
MET14	1420 (1265)	3990	1261 (1584)	612	1712 (1261)	520	1072 (1009)	915	1134 (1064)	705	1610 (1409)	1238

*Note* The total column depicts the mean/SD across all twin pairs, whereas ensuing columns stratify results per zygosity: MZM, monozygotic male twins; DZM, dizygotic male twins; MZF, monozygotic female twins; DZF, dizygotic female twins; DOS, dizygotic opposite-sex twins. **N** is the number of complete twin pairs, i.e. pairs without missing data in either twin or co-twin. MD-FS, the factor score of motor development before age 2; GM5, gross motor development at age 5; MET7, MET10, MET12, and MET14 represent the exercise behavior at each age (7, 10, 12, and 14 years)

### Phenotypic associations

Table 2 presents the phenotypic correlation matrix for early motor development and future exercise behavior traits. In the total sample, the correlation between motor milestones achievement at age 2 and gross motor competence at age 5 was 0.18. The correlations between exercise behavior at the four different ages traits ranged from 0.21 to 0.47. As shown in Table 2, similar correlations were obtained in samples of male and female twins.

In keeping with the hypothesis that motor competence developed at the early age predicts exercise level during leisure time in childhood and early adolescence, the motor development traits, MD-FS and GM5, were negatively correlated with all the exercise behavior traits, i.e., an earlier age of motor development predicted more exercise behavior. However, the effect sizes were small with median correlation of −0.10 in males, ranging from − 0.16 between MD-FS and MET7 to −0.04 between GM5 and MET10. Smaller effect sizes were seen in the females, with a mean correlation of −0.07, ranging from − 0.11 between GM5 and MET10 to −0.02 between MD-FS and MET7. For both males and females, gross motor development at age 5 was a better predictor of future exercise behavior than motor milestones attainment at age 2 (see Table 2).

**Table 2:** Phenotypic associations between early motor development and later exercise behavior

Total	MD-FS	GM5	MET7	MET10	MET12	MET14
MD-FS	—	27,135	5844	6104	11,810	7262
GM5	**0.21** ***	—	4525	4840	10,553	6373
MET7	−0.04 **	−0.09 ***	—	1736	1900	1295
MET10	**−0.06** ***	−0.11 ***	0.44 ***	—	2706	1240
MET12	**−0.07** ***	−0.10 ***	0.33 ***	0.49 ***	—	5328
MET14	**−0.09** ***	−0.10 ***	0.21 ***	0.31 ***	0.47 ***	—
**Males**	**MD-FS**	**GM5**	**MET7**	**MET10**	**MET12**	**MET14**
MD-FS	—	13,510	2997	3218	5968	3193
GM5	**0.22** ***	—	2305	2535	5321	2825
MET7	−0.05 **	−0.12 ***	—	956	979	593
MET10	**−0.08** ***	−0.14 ***	0.44 ***	—	1446	559
MET12	**−0.07** ***	−0.12 ***	0.39 ***	0.50 ***	—	2461
MET14	**−0.11** ***	−0.10 ***	0.29***	0.26 ***	0.43 ***	—
**Females**	**MD-FS**	**GM5**	**MET7**	**MET10**	**MET12**	**MET14**
MD-FS	—	13,625	2847	2886	5842	4069
GM5	**0.21** ***	—	2220	2305	5232	3548
MET7	−0.02	−0.05 **	—	780	921	702
MET10	−0.03	−0.08 ***	0.45***	—	1260	681
MET12	**−0.07** ***	−0.09 ***	0.28***	0.49***	—	2867
MET14	**−0.08** ***	−0.10 ***	0.16 ***	0.34 ***	0.50 ***	—

*Note* Associations are presented below the diagonal as Spearman rank order correlations. Corresponding sample sizes are presented above the diagonal. MD-FS, the factor score of motor development before age 2; GM5, gross motor development at age 5; MET7, MET10, MET12, and MET14 represent the exercise behavior at each age (7, 10, 12, and 14 years).

*** *p* < 0.001

The results of the phenotypic regression analyses (see Fig. 1) are summarized in Table 3, which includes the standardized regression coefficients of the exercise behaviors at the four ages on the two early motor development traits, and the proportion of explained phenotypic variance of the exercise behaviors (for non-parametric correlations, see Supplementary Table 2). In males, we note a consistent significant prediction of all exercise behavior traits by early motor development (β’s MD-FS: −0.09 to −0.04 and GM5: −0.13 to −0.11) Overall, both early motor development variables explained 2.97%, 4.05%, 2.80%, and 4.12% (all *p*’s < 001) of the phenotypic variance of exercise behavior in males at age 7, age 10, age 12, and age 14, respectively. In females, the strength of the prediction of the exercise behavior traits by early motor development was lower than in males (β’s MD-FS: −0.06 to −0.03 and GM5: −0.09 to −0.06), with female estimates for GM5 often below the lower limit of the 95% confidence intervals around the male estimates. In keeping, both early motor development variables explained only 0.48% (*p* < 0.01), 0.83% (*p* < 0.001), 1.09% (*p* < 0.001), and 1.47% (*p* < 0.001) of the phenotypic variance of exercise behavior in females at age 7, age 10, age 12, and age 14, respectively.

**Table 3:** Prediction of childhood and adolescent exercise behavior by early motor development in males and females

	Univariable regression				Multivariable regression	
Traits	MD-FS		GM5		Proportion explained (%) by MD-FS & GM5	
	Males	Females	Males	Females	Males	Females
MET7	−0.040 (−0.082, −0.001)	−0.026 (−0.069 0.018)	−0.124 (−0.163, −0.112)	−0.060 (−0.105 −0.014)	2.97	0.48
MET10	−0.062 (−0.101, −0.022)	−0.033 (−0.074 0.008)	−0.133 (−0.140, −0.127)	−0.079 (−0.089 −0.038)	4.05	0.83
MET12	−0.053 (−0.083, −0.023)	−0.047 (−0.078 −0.043)	−0.110 (−0.114, −0.105)	−0.080 (−0.085 −0.052)	2.80	1.09
MET14	−0.094 (−0.133, −0.054)	−0.055 (−0.090 −0.019)	−0.108 (−0.112, −0.073)	−0.093 (−0.125 −0.060)	4.12	1.47

*Note* Univariable standardized regression coefficients with 95% CI (columns 2–6) and joint multivariable explained variance (columns 7–8). MD-FS, the factor score of motor development before age 2; GM5, gross motor development at age 5; MET7, MET10, MET12, and MET14 represent the exercise behavior at each age (7, 10, 12, and 14 years)

### Twin correlations

**Fig. 4:**
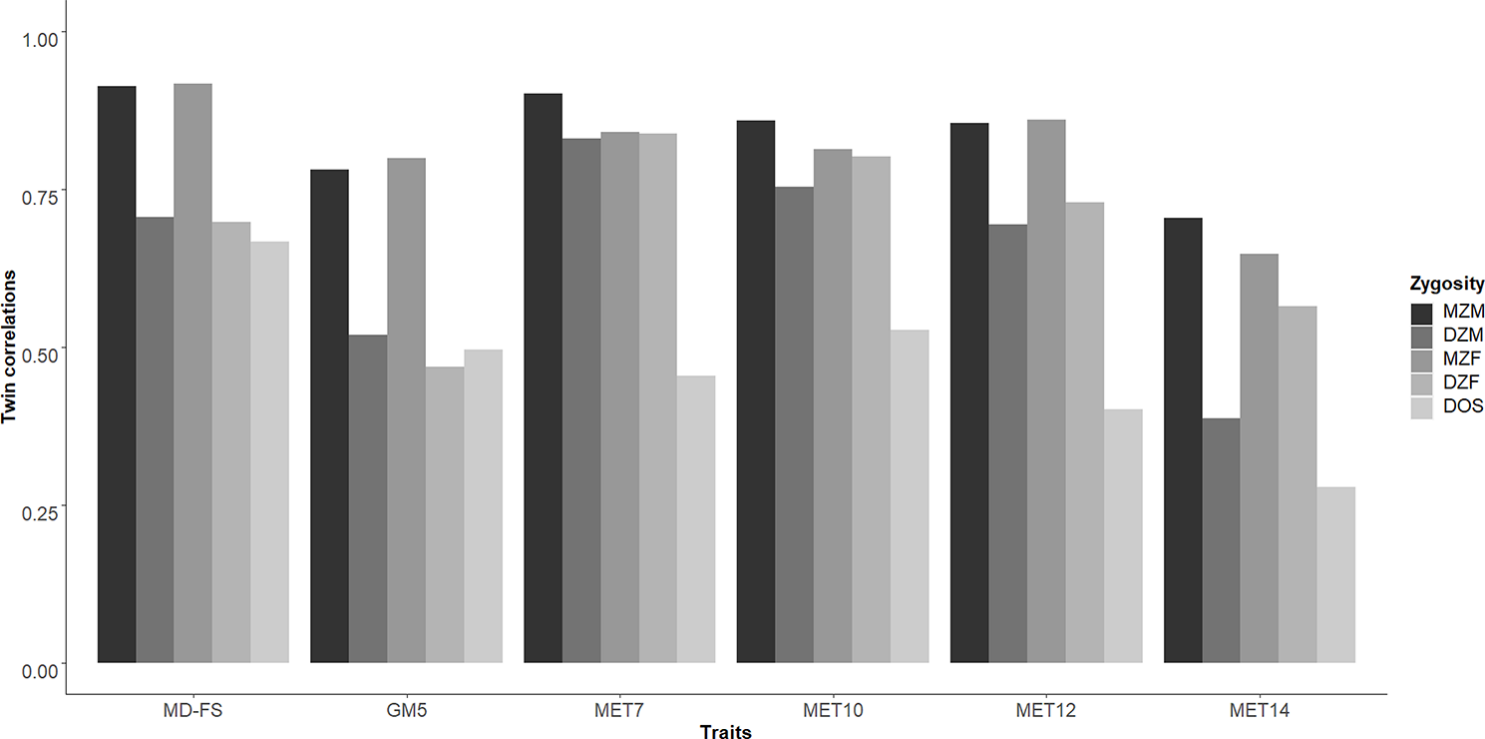
Twin correlations of early motor development and later exercise behavior measures, across the five zygosity groups *Note*: MD-FS, the factor score of motor development before age 2; GM5, gross motor development at age 5; MET7, MET10, MET12, and MET14 represent the voluntary exercise behavior at each age (7, 10, 12, and 14 years). MZM, monozygotic male twins; DZM, dizygotic male twins; MZF, monozygotic female twins; DZF, dizygotic female twins; DOS, dizygotic opposite-sex twins

The Pearson correlations for the individual early motor development and exercise behavior traits in the same-sex and opposite sex twin pairs are shown in Fig. 4 (For non-parametric correlations, see Supplementary Table 3 that also includes the complete twin pair count per trait in each zygosity group). The MZ resemblance for all traits was higher than the DZ resemblance. However, we note that the MZ correlations are less than twice the DZ correlation, which suggests (see [49]) that both genetic and shared environmental effects contribute to phenotypic variance in early motor development and leisure time exercise behavior. Lower opposite sex than same sex correlations suggest that different genetic/environmental factors may be at play in males and females. This lends support to the sex-stratified analyses.

### Multivariate genetic analysis

The relative contribution of genetic and environmental influences to the variance in early motor development and later exercise behavior traits as extracted from the multivariate Cholesky decomposition (the six-variate ACE model in Fig. 2) are shown in Table 4. For motor milestones achievement at age 2, the heritability was estimated to be 43% for males and 44% females, respectively. The shared environment explained 48% for males and 48% for females, leaving 9% and 8% of variance explained by unique environment. Our sex-stratified analyses do not allow for a direct test of quantitative sex differences in motor development, but inspection of the 95% confidence intervals suggests there were none.

For gross motor skills at age 5, sex differences did emerge. The heritability was estimated to be 57% for males and a higher estimate of 65% was found for females. The shared environment explained 23% and 16% of the variance in males and females. Again judging by the 95% confidence intervals, no sex differences in the unique environmental factors were found. These explained 20% of the variance in males and 19% of the variance in females.

The relative influence of genetic and environmental factors on exercise behavior showed a clear change over age in both sexes, but the estimates were substantially different in males and females. In males, heritability of the total weekly MET of physical activity during leisure time increased from 23% at age 7 to 51% at age 14. In parallel, the influence of the environment shared by the twins explained reduced from 68% at age 7 to 19% at age 14, whereas the influence of environmental factors unique to each twin increased from 9 to 30%. In females, heritability of the total weekly MET of physical activity during leisure time increased from 3% at age 7 to 18% at age 14. In parallel, the influence of the environment shared by the twins explained reduced from 80% at age 7 to 48% at age 14, remaining much higher than in males. As in males, the influence of environmental factors unique to each twin increased with age, from 16% at age 7 to 34% at age 14.

**Table 4:** Genetic, and shared and unique environmental influences on early motor development and later exercise behavior

Traits	Estimated variance components (%) (95%CI)					
	A_m_	C_m_	E_m_	A_f_	C_f_	E_f_
MD-FS	43.0 (40.1–46.0)	48.1 (45.1–51.0)	8.9 (8.5–9.3)	43.7 (40.7–46.8)	48.0 (44.8–50.9)	8.3 (7.9–8.8)
GM5	56.8 (51.8–62.1)	23.4 (18.4–28.2)	19.7 (18.6–20.9)	65.1 (59.5–70.9)	16.0 (10.3–21.4)	18.9 (17.9–20.0)
MET7	22.6 (18.1–27.6)	68.3 (59.6–72.6)	9.1 (8.1–10.2)	3.2 (0.1–8.0)	80.6 (76.3–83.7)	16.2 (14.5–18.1)
MET10	18.9 (13.2–25.2)	65.6 (59.6–70.8)	15.5 (13.9–17.3)	9.9 (4.3–16.2)	73.3 (67.6–78.1)	16.8 (15.0–18.9)
MET12	30.5 (25.3–36.1)	54.4 (49.1–59.3)	15.0 (13.8–16.3)	29.4 (24.4–34.9)	57.2 (51.9–62.0)	13.4 (12.3–14.5)
MET14	50.7 (36.1–64.1)	19.0 (6.6–32.4)	30.3 (26.9–34.1)	18.2 (8.1–28.9)	47.5 (37.8–56.1)	34.4 (31.3–37.8)

*Note* Standardized estimates for the contribution of genetic (A), shared environmental (C), and unique environmental (E) factors to the total variance in the traits, based on the six-variate ACE Cholesky decomposition. A_m_, C_m_, E_m_ are the genetic, shared environmental, and unique environmental influences for males, and A_f_, C_f_, and E_f_ are those for females. The A contributions correspond to ‘heritability’, i.e. heritability of MD-FS in males is 43%. MD-FS, the factor score of motor development before age 2; GM5, gross motor development at age 5; MET7, MET10, MET12, and MET14 represent the exercise behavior at each age (7, 10, 12, and 14 years). 95%CI: two-sided 95% confidence interval.

### Genetic and environmental regression of exercise behavior on motor development

From the multivariate ACE Cholesky model (see Fig. 2), we extracted the genetic, shared environmental, and unique environmental correlations, which are presented in Table 5. The pattern of correlations between the latent factors for the six traits closely resembles the pattern of the phenotypic associations. Motor milestones achievement at age 2 has a genetic correlation of 0.27 (males) and 0.32 (females) with gross motor competence at age 5, suggesting partly overlapping genetic factors influence the motor development at these two ages. Compared to motor milestones achievement, gross motor competence at age 5 has higher genetic correlations with the MET scores at all four ages (ranging from − 0.24 to −0.17 in males and − 0.20 to −0.04 in females), again suggesting motor development at age 5 to be a better predictor of exercise behavior than early motor milestone attainment. The four exercise behavior traits are significantly genetically corelated amongst each other in both males and females with higher correlations at later ages and, as expected, among measurements in closer temporal proximity. This suggest that the tracking of exercise behavior over time is largely caused by stable genetic factors.

**Table 5:** Genetic, and shared and unique environmental correlations between early motor development and later exercise behavior

	MD-FS	GM5	MET7	MET10	MET12	MET14
**1. Number of complete twin pairs**						
MD-FS	—	4567	897	903	1908	1293
GM5	4516	—	855	883	1998	1326
MET7	1001	937	—	277	321	235
MET10	1054	998	399	—	486	254
MET12	1968	2044	375	577	—	1069
MET14	941	973	200	178	872	—
**2. Genetic correlations**						
MD-FS	—	0.32*	−0.10	−0.15	−0.04	−0.14
GM5	0.27*	—	−0.15	−0.20	−0.10	−0.04
MET7	−0.01	−0.17*	—	0.88*	0.38	0.97*
MET10	−0.15*	−0.20*	0.18	—	0.77*	0.94*
MET12	−0.06	−0.24*	0.36*	0.97*	—	0.55*
MET14	−0.23*	−0.24*	0.12	0.52*	0.61*	—
**3. Shared environmental correlations**						
MD-FS	—	0.05	−0.03	−0.03	−0.07	−0.03
GM5	0.16*	—	−0.10	−0.08	−0.14	−0.29
MET7	−0.10	−0.14	—	0.52*	0.36*	0.12
MET10	−0.07	−0.17	0.54*	—	0.48*	0.40*
MET12	−0.10	−0.04	0.54*	0.40*	—	0.61*
MET14	0.00	0.00	0.82*	0.27	0.49*	—
**4. Unique environmental correlations**						
MD-FS	—	0.31*	−0.05	−0.02	−0.10	−0.12
GM5	0.33*	—	−0.04	−0.05	−0.03	−0.05
MET7	−0.13*	−0.10*	—	0.11	0.15*	0.08
MET10	−0.08	−0.10	0.46*	—	0.22*	0.00
MET12	−0.08*	−0.05	0.10	0.06	—	0.40*
MET14	−0.07	0.00	0.03	0.06	0.24*	—

*Note* Genetic, shared environmental and unique environmental correlations extracted from the ACE Cholesky model. First panel shows the number of complete twin pairs on which the correlations were based; male N below the diagonal and female N above the diagonal. Panel two shows the male genetic correlations below the diagonal and the female genetic correlations above the diagonal. Panel three shows the male shared environmental correlations below the diagonal and the female shared environmental correlations above the diagonal. Panel four shows the male unique environmental correlations below the diagonal and the female unique environmental correlations above the diagonal. MD-FS represents the factor score of motor milestones achievement before age 2; GM5 represents gross motor development at age 5; MET7, MET10, MET12, and MET14 are the exercise behavior at each age (7, 10, 12, and 14 years old).

* *p* < 0.025

Both the shared and unique environmental correlations between motor milestones and gross motor competence on the one hand and the exercise behaviors at age 7 to 14 on the other were lower than the genetic correlations, and largely non-significant. This suggests that the largest risk for confounding in the phenotypic regression results stems from genetic factors. Formal testing corroborated this, in particular for the boys. Table 6 shows the model fitting indices for the full ACE regression model and those of sub-models fixing the regression coefficients between the genetic, shared environmental and/or unique environmental factors of motor development and exercise behaviors to zero. The genetic part of the regression could not be removed from the model in boys. Fixing the relevant genetic regression coefficients resulted in a severe deterioration of the fit to the data, implying that the phenotypic regression relations are largely a reflection of the underlying genetic relations. In contrast, the shared environmental regression coefficients between early motor and later exercise behavior could each be equated to zero and they could even be deleted simultaneously with the unique environmental regression coefficients without significant loss of fit. In this model (labeled βC = 0 & βE = 0 in Table 6), the genetic regression explained 3–5% of the phenotypic variance in the male exercise behaviors at ages 7–14. Because a causal model predicts all latent factors (A, C, and E) to be correlated through the causal path, the results found in boys are not consistent with the causal model. As mentioned, genetic factors seem to explain the found phenotypic association between early motor development and later exercise behavior.

In females, both genetic and shared environmental regression coefficients could each be equated to zero separately, but this could not be done at the same time. Again the shared and unique environmental regression coefficients could be set to zero without substantial loss of fit to the data. In this model (labeled βC = 0 & βE = 0 in Table 6), the genetic regression explained 0.5–2% of the phenotypic variance in female exercise behaviors at ages 7–14. Because a causal model predicts all latent factors to be correlated through the causal path, the results found in females are again not consistent with the causal model. Rather, familial (either genetic or shared environmental) factors seems to explain the weak phenotypic association between early motor and later exercise behavior found in females.

**Table 6:** Model fit indices of the ACE regression models between later exercise behavior and early motor development

Baseline Model	Model	Model fit indices				
		−2LL	AIC	χ^2^	∆df	*p-*value
**Males**						
	ACE regression model	156338.7	156488.7	—	—	—
ACE regression model	ACE regression model, βA = 0, βC = 0, βE = 0	156578.6	156680.6	239.9	24	1.6e-37*
ACE regression model	ACE regression model, βA = 0	156367.1	156501.1	28.4	8	0.0004*
ACE regression model	ACE regression model, βC = 0	156350.5	156484.5	11.8	8	0.158
ACE regression model	ACE regression model, βE = 0	156358.4	156492.4	19.7	8	0.013
ACE regression model	ACE regression model, βC = 0 & βE = 0	156366.2	156484.2	27.5	16	0.036
ACE regression model	ACE regression model, βC = 0 & βA = 0	156495.7	156613.7	157.0	16	3.2e-25*
**Females**						
	ACE regression model	154148.9	154298.9	—	—	—
ACE regression model	ACE regression model, βA = 0, βC = 0, βE = 0	154268.4	154370.4	119.5	24	1.2e-14*
ACE regression model	ACE regression model, βA = 0	154156.0	154290.0	7.15	8	0.23
ACE regression model	ACE regression model, βC = 0	154156.8	154290.8	7.90	8	0.44
ACE regression model	ACE regression model, βE = 0	154168.8	154302.8	19.95	8	0.010*
ACE regression model	ACE regression model, βC = 0 & βE = 0	154174.7	154292.7	25.80	16	0.057
ACE regression model	ACE regression model, βC = 0 & βA = 0	154215.8	154333.8	66.93	16	3.4e-08*

*Note* βA, βC, and βE represents the genetic (βA), shared environmental (βC), and unique environmental (βE) regression of exercise behaviors on early motor development. Submodels set various regression coefficients to zero. For example, βA = 0 is the model with the regression of the latent A factors for the exercise behaviors on the latent A’s for motor milestone achievement before age 2 and gross motor competence at age 5 set to zero.

* *p* < 0.01

## Discussion

Using longitudinal data across a 12-year time span in a large population-based sample of MZ and DZ twins, we examined the strength of the association between early motor development (the age of achieving motor milestones before age 2 and gross motor competence at age 5) and exercise behavior in children and early adolescents (weekly total METminutes spent on leisure time exercise at age 7, 10, 12, and 14). We further investigated whether the association reflected a causal effect of the early motor development on future exercise behavior, after taking into account confounding by familial effects. We find evidence for a significant but weak association with early motor development accounting for about 0.5–4% of the variance in future exercise behavior in childhood and early adolescence. The association was stronger in boys than in girls. The results do not support a causal effect in either sex. Particularly in boys, the association appears to be explained largely by genetic factors that independently influenced both motor development and exercise behavior.

Substantial twin resemblance was found in boys and girls for the early motor development due to both genetic and shared environmental effects. The importance of genetic and shared (family) environmental factors for the timing of motor milestones achievement replicates our previous results in a larger sample [51]. The heritability of gross motor skills in young children has been rarely reported before and was limited to balancing ability for which a 46–62% heritability was found [35, 38]. Here, we showed that individual differences in the mother-reported mastery of 7 gross motor skills at age 5 were more heritable than motor milestones attainment in both male (57% vs. 43%) and female (65% vs. 44%) children. Shared environmental factors still played a major role in gross motor competence at age 5, but the relative contribution to the total variance was only half to one-third of that for motor milestone achievement at age 2 (23% vs. 48% for boys, 16% vs. 48% for girls).

The relative influence of genetic and shared environmental influences on exercise behavior showed a sex-specific pattern of change across age. In boys, heritability of MET increased from 23% at age 7 to 51% at age 14. In parallel, the influence of the environment shared by the twins explained reduced from 68% at age 7 to 19% at age 14. In girls, the influence of the environment shared by the twins was overall much stronger but also decreased from 80% at age 7 to 48% at age 14. In parallel, heritability increased from 3% at age 7 to 18% at age 14. These developmental changes in the genetic architecture of exercise behaviors are consistent with earlier findings [30, 37, 52]. The finding that shared environmental factors play a more prominent role in childhood than adolescence is consistent with a diminishing influence of the parents on the exercise behavior as they grow older. When the children are young, the parental influence is large because the parents provide the encouragement and the means (transport, equipment, etc.) to engage in exercise activities. Across adolescence, parental influence wanes and adolescents increasingly rely on their intrinsic motivation and on their social networks with peers to shape their behaviors. During this time, genetic effects become increasingly more prominent in explaining individual differences in regular exercise behavior. This increase in heritability may reflect genetic differences in the neurobiology of the affective responses to exercise (‘enjoyment’) as well as genetic differences in exercise abilities (‘skills’), both true and perceived [18, 53].

Our finding of a positive association between motor milestones achievement in infancy and gross motor skills at age 5 years is in accordance with a previous report [54], and suggests that motor competence is a partly stable trait. The finding that both early motor development traits significantly predicted the subsequent exercise behavior in childhood and early adolescence is consistent with previous studies [12, 55–58]. However, the significant contribution of genetic and shared environmental factors to early motor development on the one hand and childhood and adolescent exercise behavior on the other hand calls for caution in interpreting this prospective association. It supports a conservative explanation for the association between early motor development and later exercise behavior: that it is caused by these confounding familial factors. In the extant literature, most authors have declared support for the alternative explanation: that the association between early motor development and later exercise behavior reflects a causal effect of the former on the latter [8, 14–16]. However, such support was often nuanced by an expressed concern on the lack of longitudinal data and unmeasured confounding [8, 14–16].

Here, we use a genetically informative design to test a critical assumption of the causal hypothesis, namely that all latent factors influencing early motor development should also, through the causal path, influence later exercise behavior. As we find evidence for significant effects of genetic, and shared and unique environmental effects on all traits, this assumption can be restated as the expectation that the correlations between the latent genetic and shared and unique environmental factors influencing the motor development traits (MD-FS and GM5) and the subsequent exercise behavior traits (MET7, MET10, MET12, MET14) are all non-zero. A multivariate analysis separately testing the genetic and environmental parts of the phenotypic regression suggested that the causal hypothesis does not hold. In boys, we find evidence for a significant overlap in the genetic factors influencing early motor development and future exercise behavior, whereas the regression between the shared and unique environmental factors were not significant. In girls, neither genetic nor shared and unique environmental regressions were significant, likely reflecting the weaker phenotypic association, but shared familial regressors (A + C) could not be simultaneously equated to zero, suggesting familial confounding here too.

It is important to stress that the results of any observational study pertain to the situation ‘as it is now’ in the sample used for the study, not to what ‘it could be’ if large scale interventions were put in place to increase early childhood motor competence in that same sample. Put differently, the absence of evidence for a causal effect in an observational prospective study tells us that the association as encountered in the current population is not due to a direct causal effect of the predictor at the earlier timepoints on the dependents at a later time point. Our results also caution not to extrapolate the effect sizes obtained from prospective regression in observational studies to a predicted effect size of an intervention. However, establishing that causal effects are not underlying the association as encountered in the current population does not rule out that introducing an active intervention could have favorable effects. A similar mistake is often made by declaring that (high) heritability of a trait somehow signals that it is impervious to intervention. This is incorrect, as is demonstrated by, e.g., the treatment of hypertension or type II diabetes [18]. In keeping, active interventions on early children’s motor skills have been shown to facilitate motor skills at subsequent ages and to increase physical activity and sports participation [59–61].

Apart from its genetically informative design and the long follow-up time, a major strength of this study is the large sample size, as it allowed us to compute (sex-specific) estimates with narrow confidence intervals, and was instrumental for the ability to fit our multivariate models on causality and confounding. These strengths were accompanied by a number of limitations. We used the mother as the sole informant on motor development as well as the children’s exercise behaviors in leisure time. This mother report comes with a potential for bias. Mothers may over- or underestimate their children’s behavior due to social desirability or poor recall, or other biases inherent in subjective reporting. Also, they may increase/decrease the resemblance of the twin based on the perceived social desirability of the twins being unique versus similar. However, motor development is one of the more objective changes during infancy [62] and we used in part a prospective approach in which mothers were sent a memory aid in the months after birth that increases reliability [44]. Even so, a better approach would have been to use either observational instruments to assess motor development or even actual experimental motor skill testing [63, 64]. Likewise, whereas organized sports and exercise activities are considered relatively protected from recall bias by their saliency [18], confirmation of the actual intensity level, frequency and duration by accelerometers would have been more ideal and is feasible in the 7–14 age range [65]. Such methods are not easily achieved in behavioral genetic designs, given the need for a large twin sample to perform multivariate modeling of weak phenotypic associations. For similar reasons, we did not asses physical fitness traits at ages 7 to 14 which, in the theoretical framework of Stodden and colleagues, is considered a mediator of the effects of early motor development on children’s physical activity [13].

Another potential limitation, inherent to the design of the study, is the use of twin individuals who, related to their rapid catch-up trajectory to make up for the low birth-weight, may show deviant motor development compared to non-twins. However, the results of previous studies showed that the motor milestone development [66] and exercise behavior [18] in twins can be fully generalized to singleton populations.

## Conclusion

Rapid early motor development significantly predicts a larger volume of leisure time exercise behavior at the ages 7 to 14, but the effect is weak, particularly in girls. In boys, where the effect is stronger, early motor development appears to be an expression of the same genetic factors that underlie the heritability of childhood an early adolescent exercise behavior. Overall, our results do not support a direct causal effect of early motor skills on future exercise behavior.

### Electronic supplementary material

Below is the link to the electronic supplementary material.

Supplementary Material 1

Supplementary Material 2

Supplementary Material 3

## Data Availability

The datasets analyzed during the current study are available in the Netherlands Twin Registry (NTR, https://tweelingenregister.vu.nl). The datasets used during the current study are available from the NTR committee for reasonable request.

## Abbreviations

A: Genetic variance or effects
ACE: An ACE model decomposes the trait variance into additive genetic (A), common or shared environmental (C), and nonshared environmental (E) variances
C: Common or shared environmental variance or effects
DNA: Deoxyribonucleic Acid
DZ: Dizygotic Twins
DZOS: Dizygotic twins with opposite sex
E: Unique or nonshared environmental variance or effects
GM5: The Gross Motor competence at age 5
MD-FS: The early Motor Development Factor Score
MET: Metabolic Equivalents of Task
MET7: Weekly total MET spent on leisure-time physical activity at age 7
MET10: Weekly total MET spent on leisure-time physical activity at age 10
MET12: Weekly total MET spent on leisure-time physical activity at age 12
MET14: Weekly total MET spent on leisure-time physical activity at age 14
MZ: Monozygotic twins
NTR: The Netherlands Twin Register
