# Supplementary material for: Genetic confounding in the association of early motor development with childhood and adolescent exercise behavior
Source: Int J Behav Nutr Phys Act. 2024 Mar 21;21:33. doi: 10.1186/s12966-024-01583-w (PMC10958919; doi:10.1186/s12966-024-01583-w)
Supplement: Supplementary file 1 — Supplementary Material 1 [file 12966_2024_1583_MOESM1_ESM.docx]

**Participants Recruitment**

The study involved twin families registered in the Netherlands Twin Register (NTR) between 1986 and 2016. The NTR is a large-scale population-based cohort that has been recruiting and continues to recruit families with new-born twins for over 35 years. After the twin is born, parents are asked to register in the NTR, and consent to be invited for participation in research on these twins. After registration and consent of the parents, the mothers received a survey in the months after registration about the course of pregnancy, the twins’ birth, and early developmental characteristics. This first wave of data collection is followed by surveys at intervals of 2 to 3 years, always including the mothers and often also the fathers. At each wave a number of behavioral and developmental traits of the twin are repeatedly assessed and, new age-appropriate traits are assessed at specific waves. At all ages, the biological mothers report on the twins. At many ages father reports (or other live partners) on the traits are also available. After age 12, children self-report on their health and behavior. At age 16 they are asked to register in the adult NTR, which itself also sends out repeated surveys on demographics, behavioral traits, health behaviors, psychopathology, and health and wellbeing.

**The Representativeness of the Sample**

From 1986 to 2016, about 40% of all annually new born Dutch twin pairs registered in the NTR (CBS; <https://www.cbs.nl/en-gb/figures/detail/37422eng?q=twins/>).

**Difference between the Analyzed Sample and Recruited Sample**

The Netherlands Twin Register follows up all the registered twins from new-born babies until adulthood, but participation in each new wave is entirely voluntary and there is attrition over the years. We reach out to all of the original twin families at each new wave, unless they have explicitly deregistered. Analyzed sample therefore always represent a sparse matrix, for example, we may have data in one family on age 1, 3, 5, 7, 9,12,14, 16, 18, but in another family, it may be 1, 7, 12, or 1, 3, 5, 7, 9, and so on.

**Missing data strategy**

For the early motor milestones achieved before age 2, when only one single motor milestone (5 items in total) was missing, we substituted the missing motor milestone with the sample mean, thus increasing the available sample size.

For the gross motor competence at age 5 (7 items in total), one or more items were missing – for these twins the gross motor development score (the summation of 7 items score) was set to missing.

For the leisure-time physical activity, the weekly total MET values were set to missing if the question on engagement in sports activities was endorsed but none of the questions on the type(s) of activity the children engaged in was filled out. If the question on engagement in sports activities was endorsed and at least one of the questions on the type(s) of activity was filled out, any missing values for the times-per-week frequency or the duration per time were imputed using the sex/age stratified median values. This happened in ~2.5% of the sample.
