# Supplementary material for: Genetic confounding in the association of early motor development with childhood and adolescent exercise behavior
Source: Int J Behav Nutr Phys Act. 2024 Mar 21;21:33. doi: 10.1186/s12966-024-01583-w (PMC10958919; doi:10.1186/s12966-024-01583-w)
Supplement: Supplementary file 2 — Supplementary Material 2 [file 12966_2024_1583_MOESM2_ESM.doc]

STROBE Statement—Checklist of items that should be included in reports of ***cohort studies***

|  | Item No | Recommendation | Where this was addressed by Zi et al. |
| --- | --- | --- | --- |
| **Title and abstract** | 1 | (a) Indicate the study’s design with a commonly used term in the title or the abstract | Title introduces the observational and prospective nature of the study |
|  |  | (b) Provide in the abstract an informative and balanced summary of what was done and what was found | See Methods/results sections in the abstract in lines 22-45 |
| Introduction | | |  |
| Background/rationale | 2 | Explain the scientific background and rationale for the investigation being reported | Lines 85-140 |
| Objectives | 3 | State specific objectives, including any prespecified hypotheses | Lines 141-146 |
| Methods | | |  |
| Study design | 4 | Present key elements of study design early in the paper | Lines 149-152 |
| Setting | 5 | Describe the setting, locations, and relevant dates, including periods of recruitment, exposure, follow-up, and data collection | Lines 149-163 |
| Participants | 6 | (*a*) Give the eligibility criteria, and the sources and methods of selection of participants. Describe methods of follow-up | Lines 164-175 for the eligibility criteria, and methods of selection of participants. Lines 152-163 describe methods of follow-up. |
|  |  | (*b*) For matched studies, give matching criteria and number of exposed and unexposed | Lines 168-175. |
| Variables | 7 | Clearly define all outcomes, exposures, predictors, potential confounders, and effect modifiers. Give diagnostic criteria, if applicable | Lines 182, 193, and 211 |
| Data sources/ measurement | 8* | For each variable of interest, give sources of data and details of methods of assessment (measurement). Describe comparability of assessment methods if there is more than one group | Lines 182-232 |
| Bias | 9 | Describe any efforts to address potential sources of bias | Lines 154-158 describe using memory aid to decrease report bias |
| Study size | 10 | Explain how the study size was arrived at | Lines 168-175 |
| Quantitative variables | 11 | Explain how quantitative variables were handled in the analyses. If applicable, describe which groupings were chosen and why | Lines 187-192, lines 200-209, lines 226-232 describe how to handle motor milestones attainment, gross motor competence, and exercise behaviour. |
| Statistical methods | 12 | (*a*) Describe all statistical methods, including those used to control for confounding | Lines 235-287 |
|  |  | (*b*) Describe any methods used to examine subgroups and interactions | Not applicable |
|  |  | (*c*) Explain how missing data were addressed | Lines 187-190, lines 204-205 |
|  |  | (*d*) If applicable, explain how loss to follow-up was addressed | Not applicable |
|  |  | (*e*) Describe any sensitivity analyses | Not applicable |
| Results | | |  |
| Participants | 13* | (a) Report numbers of individuals at each stage of study—eg numbers potentially eligible, examined for eligibility, confirmed eligible, included in the study, completing follow-up, and analysed | Lines 290-305 |
|  |  | (b) Give reasons for non-participation at each stage | Not applicable |
|  |  | (c) Consider use of a flow diagram | Not applicable |
| Descriptive data | 14* | (a) Give characteristics of study participants (eg demographic, clinical, social) and information on exposures and potential confounders | Lines 290-305 |
|  |  | (b) Indicate number of participants with missing data for each variable of interest | Lines 187-190, lines 204-205 |
|  |  | (c) Summarise follow-up time (eg, average and total amount) | Lines 295-300 |
| Outcome data | 15* | Report numbers of outcome events or summary measures over time | Lines 295-305, and table 1 |
| Main results | 16 | (*a*) Give unadjusted estimates and, if applicable, confounder-adjusted estimates and their precision (eg, 95% confidence interval). Make clear which confounders were adjusted for and why they were included | Lines 308-420 |
|  |  | (*b*) Report category boundaries when continuous variables were categorized | Not applicable |
|  |  | (*c*) If relevant, consider translating estimates of relative risk into absolute risk for a meaningful time period | Not applicable |
| Other analyses | 17 | Report other analyses done—eg analyses of subgroups and interactions, and sensitivity analyses | Not applicable |
| Discussion | | |  |
| Key results | 18 | Summarise key results with reference to study objectives | Lines 424-435 |
| Limitations | 19 | Discuss limitations of the study, taking into account sources of potential bias or imprecision. Discuss both direction and magnitude of any potential bias | Lines 515-528 |
| Interpretation | 20 | Give a cautious overall interpretation of results considering objectives, limitations, multiplicity of analyses, results from similar studies, and other relevant evidence | Lines 436-501 |
| Generalisability | 21 | Discuss the generalisability (external validity) of the study results | Lines 526-528 |
| Other information | | |  |
| Funding | 22 | Give the source of funding and the role of the funders for the present study and, if applicable, for the original study on which the present article is based | Lines 66-73 |

*Give information separately for exposed and unexposed groups.

**Note:** An Explanation and Elaboration article discusses each checklist item and gives methodological background and published examples of transparent reporting. The STROBE checklist is best used in conjunction with this article (freely available on the Web sites of PLoS Medicine at http://www.plosmedicine.org/, Annals of Internal Medicine at http://www.annals.org/, and Epidemiology at http://www.epidem.com/). Information on the STROBE Initiative is available at http://www.strobe-statement.org.
