# Supplementary material for: Genetic confounding in the association of early motor development with childhood and adolescent exercise behavior
Source: Int J Behav Nutr Phys Act. 2024 Mar 21;21:33. doi: 10.1186/s12966-024-01583-w (PMC10958919; doi:10.1186/s12966-024-01583-w)
Supplement: Supplementary file 3 — Supplementary Material 3 [file 12966_2024_1583_MOESM3_ESM.doc]

**Supplementary Table 1. Demographics of the twins at each age, per wave of data collection.**

| **Traits** | **N** | **% MZ twin** | **% Male** | **Age (SD)** |
| --- | --- | --- | --- | --- |
| **MD-FS** | 41822 | 33.6% | 50.0% | 2.32 (0.24) |
| **GM5** | 34378 | 33.9% | 49.9% | 5.46 (0.40) |
| **MET7** | 7264 | 37.2% | 52.7% | 7.50 (0.34) |
| **MET10** | 7470 | 37.0% | 52.5% | 9.81 (0.42) |
| **MET12** | 14086 | 36.0% | 50.5% | 12.25 (0.40) |
| **MET14** | 7980 | 30.7% | 44.0% | 14.61 (0.51) |

**Supplementary Table 2. Non-parametric correlations between early motor development and later exercise behavior.**

| **Total** | **MD-FS** | **GM5** | **MET7** | **MET10** | **MET12** | **MET14** |
| --- | --- | --- | --- | --- | --- | --- |
| MD-FS | — | 27135 | 5844 | 6104 | 11810 | 7262 |
| GM5 | **0.18** *** | — | 4525 | 4840 | 10553 | 6373 |
| MET7 | -0.03 | **-0.08 ***** | — | 1736 | 1900 | 1295 |
| MET10 | **-0.05***** | **-0.13 ***** | **0.45 ***** | — | 2706 | 1240 |
| MET12 | **-0.06** *** | **-0.09 ***** | **0.35 ***** | **0.56 ***** | — | 5328 |
| MET14 | **-0.09** *** | **-0.10 ***** | **0.27 ***** | **0.42 ***** | **0.55 ***** | — |
| **Males** | **MD-FS** | **GM5** | **MET7** | **MET10** | **MET12** | **MET14** |
| MD-FS | — | 13510 | 2997 | 3218 | 5968 | 3193 |
| GM5 | **0.18** *** | — | 2305 | 2535 | 5321 | 2825 |
| MET7 | -0.04 | **-0.11 ***** | — | 956 | 979 | 593 |
| MET10 | **-0.08***** | **-0.16 ***** | **0.47 ***** | — | 1446 | 559 |
| MET12 | **-0.06** *** | **-0.11 ***** | **0.41 ***** | **0.55 ***** | — | 2461 |
| MET14 | **-0.11** *** | **-0.10 ***** | **0.28***** | **0.41 ***** | **0.53 ***** | — |
| **Females** | **MD-FS** | **GM5** | **MET7** | **MET10** | **MET12** | **MET14** |
| MD-FS | — | 13625 | 2847 | 2886 | 5842 | 4069 |
| GM5 | **0.17** *** | — | 2220 | 2305 | 5232 | 3548 |
| MET7 | -0.02 | -0.06 | — | 780 | 921 | 702 |
| MET10 | -0.02 | **-0.11***** | **0.41***** | — | 1260 | 681 |
| MET12 | **-0.06** *** | **-0.08 ***** | **0.28***** | **0.56***** | — | 2867 |
| MET14 | **-0.07** *** | **-0.11 ***** | **0.26 ***** | **0.42 ***** | **0.54 ***** | — |

*Note:* Associations are presented below the diagonal as Spearman rank order correlations (polychoric correlation for GM5). Corresponding sample sizes are presented above the diagonal. MD-FS, the factor score of motor development before age 2; GM5, gross motor development at age 5; MET7, MET10, MET12, and MET14 represent the exercise behavior at each age (7, 10, 12, and 14 years). *** p < 0.001

**Supplementary Table 3: Non-parametric twin correlations of early motor development and exercise behavior traits.**

| **Traits** | **MD-FS** | | **GM5** | | **MET7** | | **MET10** | | **MET12** | | **MET14** | |
| --- | --- | --- | --- | --- | --- | --- | --- | --- | --- | --- | --- | --- |
|  | **Correlation** | **N** | **Correlation** | **N** | **Correlation** | **N** | **Correlation** | **N** | **Correlation** | **N** | **Correlation** | **N** |
| **MZM** | **0.93** | 3510 | **0.87** | 2914 | **0.87** | 675 | **0.85** | 691 | **0.86** | 1269 | **0.73** | 612 |
| **DZM** | **0.73** | 3527 | **0.69** | 2900 | **0.75** | 665 | **0.73** | 672 | **0.66** | 1153 | **0.42** | 520 |
| **MZF** | **0.93** | 3724 | **0.90** | 3161 | **0.80** | 618 | **0.86** | 651 | **0.87** | 1314 | **0.70** | 915 |
| **DZF** | **0.73** | 3258 | **0.70** | 2687 | **0.79** | 526 | **0.75** | 525 | **0.72** | 1040 | **0.58** | 705 |
| **DOSmf** | **0.69** | 6874 | **0.67** | 5527 | **0.44** | 1148 | **0.45** | 1196 | **0.41** | 2267 | **0.31** | 1238 |

*Note:* Correlations were based on Spearman for MD-FS, and all four MET scores and on the polychoric correlation for GM. **N** is the number of complete twin pairs, representing the number of twin pairs without missing data in both twin individuals; MD-FS, the factor score of motor development before age 2; GM5, gross motor development at age 5; MET7, MET10, MET12, and MET14 represent the voluntary exercise behavior at each age (7, 10, 12, and 14 years); MZM, monozygotic male twins; DZM, dizygotic male twins; MZF, monozygotic female twins; DZF; dizygotic female twins; DOSmf, opposite sex twin pairs (ordered male-female).
